# Association Between Premorbid Body Mass Index and Amyotrophic Lateral Sclerosis: Causal Inference Through Genetic Approaches

**DOI:** 10.3389/fneur.2019.00543

**Published:** 2019-05-24

**Authors:** Ping Zeng, Xinghao Yu, Haibo Xu

**Affiliations:** ^1^Department of Epidemiology and Biostatistics, School of Public Health, Xuzhou Medical University, Xuzhou, China; ^2^Center for Medical Statistics and Data Analysis, School of Public Health, Xuzhou Medical University, Xuzhou, China

**Keywords:** body mass index, amyotrophic lateral sclerosis, Mendelian randomization, instrumental variable, genome-wide association studies

## Abstract

**Purpose:** Inverse association between premorbid body mass index (BMI) and amyotrophic lateral sclerosis (ALS) was implied in observational studies; however, whether this association is causal remains largely unknown.

**Materials and Methods:** We first conducted a meta-analysis to investigate whether there exits an association between premorbid BMI and ALS. We then employed a two-sample Mendelian randomization approach to evaluate the causal relationship of genetically increased BMI with the risk of ALS. The Mendelian randomization analysis was implemented using summary statistics for independent instruments obtained from large-scale genome-wide association studies of BMI (up to ~770,000 individuals) and ALS (up to ~81,000 individuals). The causal effect of BMI on ALS was estimated using inverse-variance weighted methods and was further validated through extensive complementary and sensitivity analyses.

**Results:** The meta-analysis showed that a unit increase of premorbid BMI can result in about 3.0% (95% CI 2.1–4.5%) risk reduction of ALS. Using 1,031 instruments that were strongly related to BMI, the causal effect of per one standard deviation increase of BMI was estimated to be 1.04 (95% CI 0.97–1.11, *p* = 0.275) in the European population. This null association between BMI and ALS also held in the East Asian population and was robust against various modeling assumptions and outlier biases. Additionally, the Egger-regression and MR-PRESSO ruled out the possibility of horizontal pleiotropic effects of instruments.

**Conclusion:** Our results do not support the causal role of genetically increased or decreased BMI on the risk of ALS.

## Introduction

Amyotrophic lateral sclerosis (ALS) is among the most frequent adult-onset fatal neurodegenerative diseases and is clinically characterized by rapidly progressive motor neurons degeneration and death because of respiratory failure (1). Although great advance has been made for the understanding of ALS in the past decades, the pathogenic mechanism underlying ALS remains largely unknown and only few therapeutic options can be available (2). It has been reported that both genetic (3, 4) and environmental factors (e.g., cigarette smoking, alcohol consumption, exposure to pesticides, lead, organic toxins or electromagnetic radiation, and socioeconomic status) may contribute to the development of ALS (5–11). However, few replicable and definitive environmental risk factors are currently well-established for ALS. In addition, due to the quickly growing population aging in the upcoming years, it is evaluated that the number of ALS cases across globe will increase by ~70% (12), which is anticipated to result in rather serious socioeconomic and health burden. Therefore, understanding the risk factors of ALS for improving the medical intervention and quality of life for ALS patients is considerably important from both disease treatment and public health perspectives.

Among extensive epidemiological researches of ALS, an important observation is that ALS patients often encounter a loss of weight or a decrease of body mass index (BMI) at the early phase of diagnosis (1, 13–20). Indeed, substantial change of BMI in ALS patients has been identified as an independent prognostic factor and has been linked to disease progression (19, 21–27). For example, it is observed that a rapid reduction of BMI in ALS patients at the initial disease stage is a strong indicator of faster disease progression and shorter survival time, consistent with the finding that nutritional intervention for ALS patients to increase BMI can prolong the survival time and lead to a delay in disease progression (28–30). The benefit of raising BMI for ALS patients by taking high-energy diet is also confirmed by mouse models (31).

However, whether the long-term exposure to genetically increased (or decreased) BMI prior to the onset of ALS also plays a pathological role in the development of ALS is less understood. Various findings with regard to the relationship between premorbid (i.e., prediagnostic) BMI and ALS have been reported in the literature. In a large-scale observational cohort, it was shown that higher BMI before the onset of ALS was associated with a decreased risk of ALS, resulting in an average of 4.6% [95% confidence interval (CI) 3.0–6.1%] lower risk of ALS per unit increase in BMI (25). This inverse association between premorbid BMI and the risk (or mortality) of ALS was also supported by recent studies (Table S1) (32–36). However, contradictory results were also reported. For example, it was shown that ALS cases consistently had a greater BMI compared with controls beyond 5 years before ALS manifestation although they had a smaller BMI than controls within 5 years prior to onset; and that per unit increase of BMI can result in ~5.0% (95% CI 0.0–11.0%) higher risk of ALS (Table S2 and Figure S1) (19). This study also implied that BMI may already begin to change about 10 years before onset of ALS (19). A more pronounced increased risk associated with greater BMI before 5 years onset of ALS was observed in a population-based case-control study performed in Washington State (37, 38): 50% higher risk of ALS for those with BMI between 24 and 26 kg/m^2^ compared with those with BMI <21 kg/m^2^, and 70% higher risk of ALS for those with BMI larger than 26 kg/m^2^ compared with those with BMI <21 kg/m^2^.

The conflicting observations on the relationship between premorbid BMI and ALS may be partly due to uncontrolled/unknown bias or confounding factors that are frequent in observational studies, or partly due to the relatively small sample size for patients because of the rarity of ALS, or partly due to the reverse causality as well as a limited retrospective time (or follow-up) before ALS onset (19). Overall, an essential problem still exists—is the change of BMI before ALS manifestation a causal risk factor or the consequence of ALS?

Because BMI is a modifiable exposure factor and obesity is a growing global health problem (39), a better characterization of the causal effect of BMI on ALS can thus facilitate our understanding of the pathogenesis of ALS and finally lead to better prevention and treatment for ALS patients. Traditionally, randomized controlled trial (RCT) studies are the gold standard for inferring the causal impact of exposure on outcome. However, determining the causal relationship between premorbid BMI and ALS through RCT is challenging and unrealistic, because RCT necessarily requires a very large set of subjects and an extremely long follow-up before clinical manifest of ALS due to its rarity in the population (40) and wide variations in prevalence and incidence across various age groups (41–44). Therefore, it is desirable to investigate the causal association between premorbid BMI and ALS through observational studies. The Mendelian randomization approach may help clarify the causal relationship (45, 46) by employing single nucleotide polymorphisms (SNPs) as instrument variables for the exposure (i.e., premorbid BMI) to assess its causal effect on the outcome of interest (i.e., ALS) (Figure S2) (47). Intuitively, we are dependent on the idea that SNPs which influence the exposure would also affect the risk of outcome through the change of exposure. The recent successes of large-scale genome-wide association studies (GWASs) (48–52) make it feasible to choose strongly associated SNPs to be valid instruments for causal inference in Mendelian randomization (53, 54). Indeed, in the last few years Mendelian randomization has become a considerably powerful method for causality inference in observational studies (53, 55).

In the present study we first conducted a meta-analysis to investigate whether there is an association between premorbid BMI and ALS. As a result, we found that an inverse association existed. Furthermore, to examine whether this negative association was causal, we conducted the largest and most comprehensive two-sample Mendelian randomization analysis to date by using summary statistics obtained from large-scale GWASs with ~770,000 individuals for BMI and ~21,000 cases for ALS.

## Materials and Methods

### Systematic Review and Meta-Analysis

We first employed the systematic review and meta-analysis to provide a pooled conclusion about the relationship between premorbid BMI and ALS. Following the PRISMA guidelines (56), we carried out a literature search mainly on the database PubMed, with the detailed search strategy given in Supplementary Text S1. For the included articles (Table 1 and Figure M1), we then extracted from the literature with the information on study setting, study design, population, and case/control, observational period as well as measurement results of effect size [odds ratio (OR), relative ratio (RR), or hazard ratio (HR)]. Based on those extracted data, we evaluated the association between premorbid BMI and the risk of ALS via inverse-variance weighted methods. Details of the meta-analysis were shown in Supplementary Text S1.

**Table 1:** Summary information of 11 studies included in the meta-analysis.

**First author** **(year)**	**Country (or region)**	**Study design**	**Fellow up period**	**Total *N*** **(case/control)**	**OR/RR/HR**	**Covariates**
Nelson et al. (37, 38)	USA	Case-control	1990–1994	482 (161/321)	1.06 (0.95–1.16)	Cases and controls were matched on age, gender, and respondent type; the conditional logistic regression was used while adjusting for education, total energy intake, and smoking.
Scarmeas et al. (32)	USA	Case-control	1992-2000	409 (270/139)	0.94 (0.90–0.99)	Age, sex, always-slim, and varsity athlete
Veldink et al. (70)	Netherlands	Case-control	2001–2002	473 (219/254)	1.02 (0.97–1.08)	Sex, age, education, smoking, and alcohol use
Sutedja et al. (33)	Netherlands	Case-control	2004–2009	872 (334/538)	0.92 (0.87–0.97)	Age and sex
Doyle et al. (71)	UK (women)	Prospective cohort	1996–2001	1.3 M (752/NA)	0.98 (0.96–0.99)	Region, deprivation, year of birth, use of hormone replacement therapy, smoking, and alcohol use
Gallo et al. (34)	Europe	Prospective cohort	1992–2002	518,108 (222/NA)	0.97 (0.94–1.01)	Sex, education, and smoking
O'Reilly et al. (25)	USA	Prospective cohort	1976–2008	1,100,910 (1,153/NA)	0.94 (0.92–0.97)	Smoking, vitamin E from food and supplements, education, and physical activity
Huisman et al. (35)	Netherlands	Case-control	2006–2011	2,767 (674/2,093)	0.97 (0.94–0.99)	Age, sex, education, smoking, lifetime physical activity, and total energy intake
Mariosa et al. (36)	USA (aged at 25 years)	Case-control	2005–2010	1,442 (467/975)	0.99 (0.95–1.03)	Age, use of VA health care, sex, race/ethnicity, smoking, and education
Mariosa et al. (36)	USA (aged at 45 years)	Case-control	2005–2010	1,442 (467/975)	0.95 (0.91–0.98)	Age, use of VA health care, sex, race/ethnicity, smoking, and education
Aberg et al. (72)	Swedish (men)	Prospective cohort	1968–2005	1,819,817 (526/NA)	0.96 (0.93–0.99)	Age
O'Reilly et al. (73)	USA, Europe, and Australia	Prospective Cohort (death)	1986–2010	568,060 (428/NA)	0.97 (0.95–0.99)	Smoking, education, physical activity, and race

### GWAS Data Sources and Instrument Selection for Mendelian Randomization

We selected independent index association SNPs (*p* < 5.00E-8) to serve as instrumental variables for BMI (Table S3) from the Genetic Investigation of ANthropometric Traits (GIANT) consortium, which is the largest BMI GWAS (up to 773,253 individuals) for the European population to date (Supplementary Text S2) (52). For all the selected instruments we obtained their association summary statistics in terms of the effect allele, marginal effect size estimate, and standard error. To estimate the causal effect of BMI on ALS, we extracted the corresponding association summary statistics of these index SNPs for ALS from the ALS Variant Server (AVS) GWAS that was also carried out in the European population up on 80,610 individuals (20,806 cases and 59,804 controls) (Supplementary Text S2) (4).

Besides the set of instruments of BMI obtained from Yengo et al. (52), as a part of complementary and sensitivity analyses, we also attempted to validate whether the relationship between BMI and ALS derived from the European population also holds in the East Asian population. To do so, we performed an additional Mendelian randomization study using another set of instruments obtained from an East Asian BMI GWAS up to 158,284 individuals (Table S4 and Supplementary Text S2) (50). The corresponding summary statistics of ALS for these instruments were extracted from an East Asian ALS GWAS up to 4,084 individuals (1,234 cases and 2,850 controls) (Supplementary Text S2) (57). The two sets of index SNPs of BMI from the two populations share only one common instrument (i.e., rs7903146). The GWAS genetic data sets used in the present study are summarized in Table 2.

**Table 2:** GWAS genetic data sets used in the Mendelian randomization analysis in the main text.

**Traits**	**PubMed ID**	**Population**	***k***	***p***	**Sample size**	**Data source**
BMI	30124842	European	1,031	2,336,269	773,253	GIANT (52)
ALS	29566793	European		10,031,417	80,610	(4)
BMI	28892062	East Asian	75	6,108,953	158,284	BioBank Japan (50)
ALS	28931804	East Asian		6,613,544	4,084	(57)

*BMI, body mass index; ALS, amyotrophic lateral sclerosis; k is the number of instruments and p is the total number of SNPs in the GWAS genetic data. Note that there were ~41,000 and ~5,000 BMI-associated SNPs (p < 5E−8) in the European and East Asian GWAS genetic data sets, the shared number of BMI-associated SNPs was ~1,100*.

### Estimation of Causal Effect With Inverse-Variance Weighted Methods

To examine whether the instruments are strong, for each index SNP that was used as instrument, we calculated the proportion of phenotypic variance of BMI explained (PVE) by the instrument using summary statistics (58) and generated the *F* statistic (Table S3) (59, 60). We then performed the two-sample Mendelian randomization analysis (61, 62) and estimated the causal effect of BMI on ALS in terms of OR per standard deviation (SD) change in BMI with inverse-variance weighted (IVW) methods (60, 63). Before formal analysis, to ensure the validity of Mendelian randomization analysis, we examined the pleiotropic associations of instruments by removing SNPs that may be associated with ALS with a marginal *p*-value below 0.05 after Bonferroni correction. In our analysis no instruments were excluded from any set of instruments by this strategy. In addition, we employed the Cochran's *Q*-test to examine the heterogeneity of causal effect across instruments (64) and performed power calculation using an analytic method (https://cnsgenomics.shinyapps.io/mRnd/) (65).

### Complementary and Sensitivity Analyses

To ensure results robustness and guard against model assumptions in the Mendelian randomization analysis (Figure S2), we carried out a series of complementary and sensitivity analyses: (i) leave-one-out (LOO) cross-validation analysis (66) and MR-PRESSO analysis (67) to validate whether there are instrumental outliers that can substantially influence the causal effect estimate; (ii) weighted median-based method that is robust when some instruments are invalid (68); (iii) MR-Egger regression to examine the assumption of directional pleiotropic effects (61, 69); (iv) IVW causal analysis after removing instruments that may be correlated to other 38 complex metabolic, anthropometric, and socioeconomic traits from large-scale GWASs (Supplementary Text S2); (v) reverse causal inference on BMI using ALS instruments; (vi) IVW method for the causal effect estimation of BMI on ALS in the East Asian population.

## Results

### Identified Association Between Premorbid BMI and ALS via Meta-Analysis

**Figure 1:**
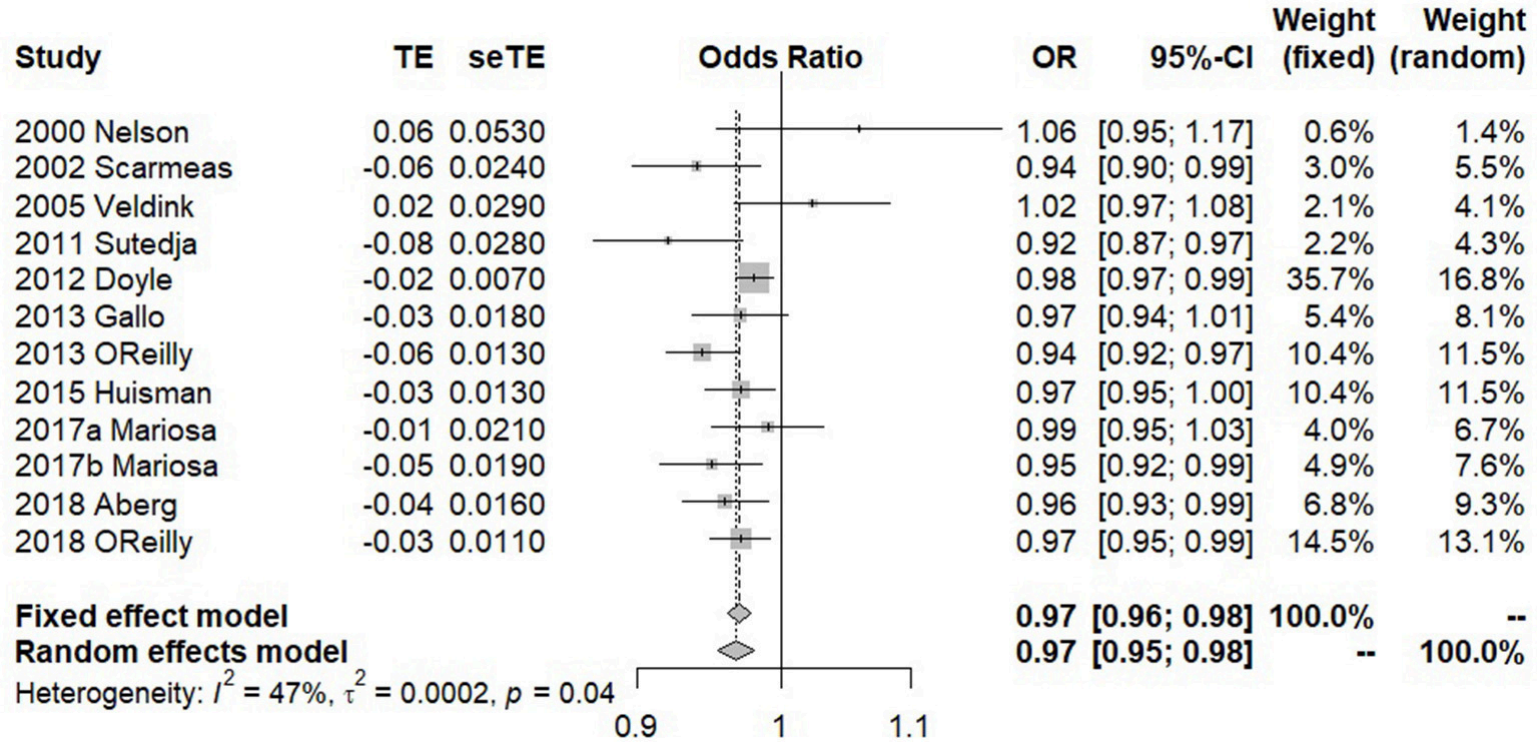
Combined OR of meta-analysis when all the 11 studies are included in our study.

In our meta-analysis, a total of 11 previous studies were finally included (Table 1). Among those studies, two showed a positive association between premorbid BMI and ALS, while the rest showed a negative relationship. The forest plot for premorbid BMI with the risk of developing ALS is displayed in Figure 1. The heterogeneous effect size of BMI on ALS in those studies is observed (the Q statistic is 20.64 with the *p*-value 0.037 and I2 is 47%). The fixed-effects model and the random-effects model generated similar pooled estimates. Specifically, for example, the random-effects model shows that a unit increase of premorbid BMI can result in about 3.0% (95% CI 2.1–4.5%) risk reduction of ALS (25, 32–36). We also found that the pooled estimate of effect size is robust against various subgroup sensitivity analyses (Figures M2–M7) and that no single study can substantially dominate the final estimate in the meta-analysis (Table M1). Additionally, the funnel plot (Figure M8) and the Egger test (*p* = 0.756) together demonstrate that the publication bias is unlikely to influence the estimate of the meta-analysis. In summary, based on the results of meta-analysis above, we can conclude that there exists a negative association between premorbid BMI and ALS.

### Causal Effect of BMI on ALS With IVW Mendelian Randomization Analyses

By using the clumping procedure of PLINK (details in Supplementary Text S2), we selected 1,031 independent SNPs to be valid instruments for BMI in the European population from the GIANT study (52) (Table S3). These instruments together explain a total of 8.28% of phenotypic variance for BMI. The *F* statistics for all these SNPs are above 10 (ranging from 28.8 to 1426.2, with an average of 58.4), implying that the weak instrument is less likely to bias our analysis. In addition, as there is significantly statistical evidence for the heterogeneity of causal effect across instruments (*p* = 7.43E-4 in terms of the Cochran's Q test); therefore, only the results estimated using the random-effects IVW method are displayed in the following paragraphs.

Specifically, we found that the OR per unit SD increase of BMI on ALS is estimated to be 1.04 (95% CI 0.97–1.11, *p* = 0.275) using the set of 1,031 instruments, indicating that the genetically changed BMI is not causally associated with an increased or decreased risk of ALS. The association is essentially unaltered if another set of instruments is employed (Supplementary Text S2). We further examine whether the lack of detectable non-zero causal effect of BMI on ALS is due to a lack of statistical power. To do so, we calculated the statistical power to detect an OR of 1.10 (or 0.90) per unit change of BMI on the risk of ALS following the analytic approach (65). In the power calculation, we set the total phenotypic variance of BMI explained by all the instrumental variables to be 8.28% (see above), the significance level α to be 0.05, and the proportion of the ALS cases to be 25.8% [i.e., the proportion of cases observed in the AVS study (4)]. It is shown that the estimated statistical power is 96% if *n* = 80,610 (i.e., the sample size of the ALS GWAS), indicating that we would have reasonably high power to detect such a causal effect of BMI on ALS if BMI is indeed causally related to the risk of ALS.

### Sensitivity Analyses to Validate the Estimated Causal Effect of BMI on ALS

**Figure 2:**
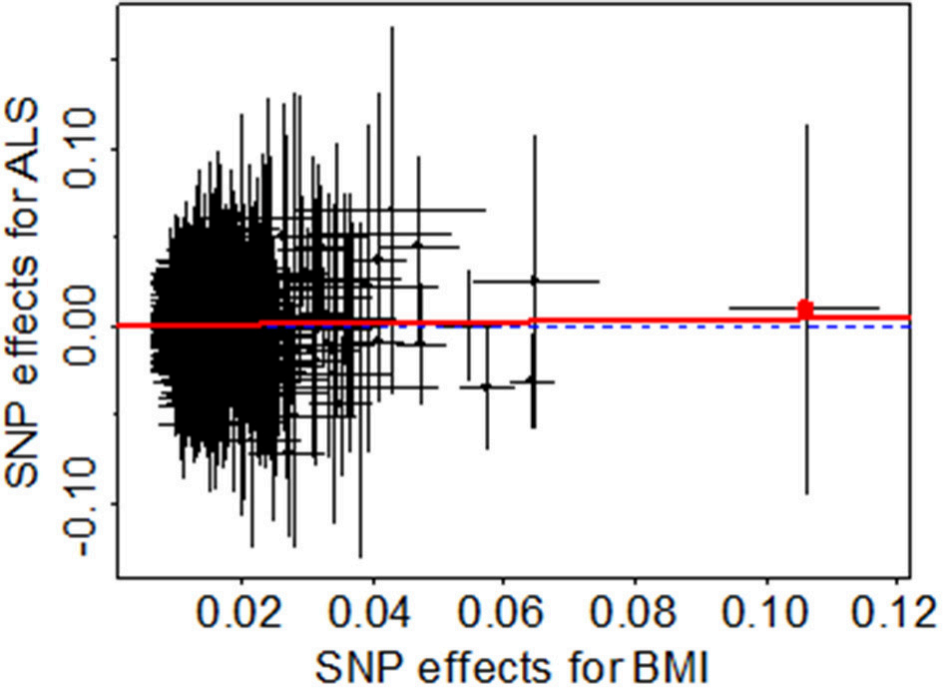
Relationship between the SNP effect size estimates of BMI (x-axis) and the effect size estimates of ALS (y-axis) in the European population using 1,031 instruments generated from Yengo et al. (52). In the plot, the 95% CIs for the effect sizes of instruments on BMI are shown as horizontal lines, while the 95% CIs for the effect sizes of instruments on ALS are shown as vertical lines. The horizontal dotted line represents zero effects. The line in red represents the estimated causal effect of BMI on ALS obtained using the random-effects IVW method. The red dot in the rightmost side is identified as an outlier (i.e., rs2229616).

**Figure 3:**
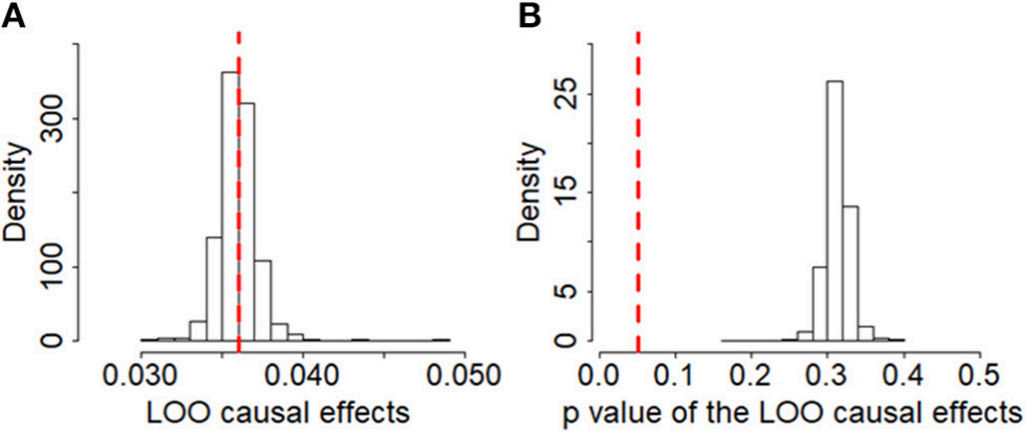
Leave-one-out (LOO) results of BMI on ALS based on the set of 1,091 instruments in the European population. **(A)** Estimated LOO causal effects; **(B)** The *p*-values of the LOO causal effects. In the left panel, the red reference line is the point estimate of causal effect for BMI using all the instruments; in the right panel, the red reference line represents the significance level of 0.05.

To guard against the potential false negative error, we now validate the null causal association between BMI and ALS identified above through various sensitivity analyses. First, we examine whether there exist potential instrument outliers and whether these outliers have a substantial influence on the estimate of causal effect. To do so, we created a scatter plot by drawing the effect sizes of BMI with regard to their effect sizes of ALS for all the 1,031 instruments. Among all the instruments, one index SNP (i.e., rs2229616) has the largest effect size of 0.106 on BMI and can be reasonably assumed to be a potential outlier (Figure 2). However, this outlier does not largely change the estimated causal effect in our analysis. Specially, after removing rs2229616, the OR per one SD increase of BMI on ALS is 1.04 (95% CI 0.97–1.11, *p* = 0.317), in line with that obtained using all the instruments. To further examine whether a single instrument may strongly influence the causal effect of BMI on ALS, we performed a leave-one-out (LOO). Again, the LOO analysis results show that no single instrument can influence the causal effect estimate substantially (Figure 3). Additionally, we also directly tested for whether any instrument is an outlier using MR-PRESSO (67), which shows that no significant instrument outliers exist in our analysis at the significance level of 0.05.

**Figure 4:**
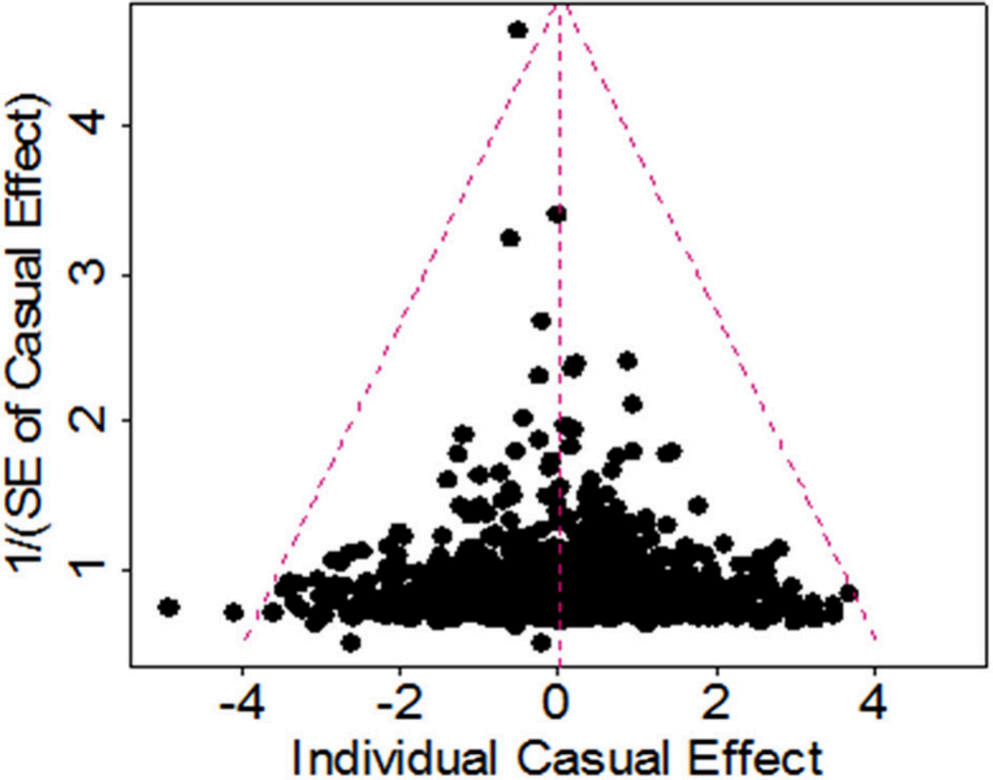
Funnel plot for single causal effect estimate of BMI on ALS obtained using all 1,091 instruments in the European population.

To study whether some are invalid among the set of 1,031 instruments and may bias the results, we conducted a Mendelian randomization analysis using the weighted median method (68). The weighted median method yields a similar estimate as before. In particular, the OR per one SD increase of BMI on ALS is 1.01 (95% CI 0.91–1.13, *p* = 0.806), suggesting that invalid instruments unlikely bias our results. To investigate whether those instruments show potentially horizontal pleiotropy, we performed the MR-Egger regression (61, 69). The results from the MR-Egger regression analysis are again largely consistent with our main results. For example, using all the 1,031 instruments the MR-Egger regression estimates the OR per one SD increase of BMI on ALS to be 0.97 (95% CI 0.79–1.19, *p* = 0.740). The MR-Egger regression intercept is 0.001 (95% CI −0.002~0.004, *p* = 0.473). Furthermore, the funnel plot also displays a symmetric pattern around the causal effect point estimate (Figure 4), which, along with the MR-Egger regression, offers no evidence for horizontal pleiotropy.

We further performed causal estimation for BMI after removing instruments that may be potentially associated with other 38 complex traits (Supplementary Text S2) with various *p*-value thresholds. Again, the resulting estimates of causal effect for BMI on ALS are not statistically significant regardless of the thresholds used, consistent with the results obtained with all the instruments (Table S5). The Mendelian randomization analysis using ALS-related instruments also removes the likelihood of reverse causality (the causal effect size is −0.011, 95% CI −0.034~0.011, *p* = 0.317). However, we emphasized this reverse causality result should be interpreted with caution since only one instrument of ALS was employed, which may be underpowered to detect the causal effect of ALS on BMI.

Finally, we estimated the causal effect of BMI on ALS in the East Asian population. To do so, we first obtained a set of 75 BMI-associated SNPs to serve as instruments (Table S4). The estimated PVE by these instruments is 2.59%, and all have an *F* statistic above 10 (range from 22.6 to 410.1, with an average of 56.8) and are thus deemed as strong instruments (59, 60). With these identified instruments, again we found that genetically higher BMI is not causally associated with an increased or decreased risk of ALS at the significance level of 0.05. Specifically, the OR per one SD increase of BMI on ALS is estimated to be 0.90 (95% CI 0.59–1.39, *p* = 0.647) in the East Asian population.

## Discussion

### Summary of the Results of Our Study

It is controversial on whether there exists a relationship between premorbid BMI and ALS in the literature. To answer this problem, in the present paper we have performed a systematic review and meta-analysis and showed that premorbid BMI is inversely associated with ALS, supporting the previous findings from epidemiological studies (Supplementary Text S1) (32–38). To further explore whether this association is causal and to investigate whether genetic predisposition to BMI plays an etiological role in ALS, we have implemented a comprehensive Mendelian randomization analysis using summary statistics from GWASs. Compared with observational studies, Mendelian randomization has the advantage that its results are not susceptible to the measurement error bias and are also less susceptible to reverse causation and confounders.

However, our Mendelian randomization analysis does not support the existence of causal association between premorbid BMI and the risk of ALS in both the European and East Asian population. We also validated that the failure of identifying non-zero causal effect of BMI on ALS is not possibly due to the lack of statistical power. To our knowledge, this is the first Mendelian randomization study to explore the relationship between BMI prior to disease onset and ALS by leveraging genetic information from large scale GWASs (74). As little has been known about the casual factors for the development of ALS to date (2); therefore, our study contributes considerably to the research on the role of premorbid BMI with regard to the ALS risk and has the potential implication in public health.

### Treatment of the Model Assumptions of Mendelian Randomization

Note that we employed a vast set of independent and strongly associated instrument variables (a total of 1,031) for causality inference of BMI on ALS. The benefit of applying multiple instruments in Mendelian randomization analysis is that the possibility of weak instruments bias is less likely and the high statistical power is guaranteed. However, it also has a high likelihood to incorporate pleiotropic instruments, which violates the assumptions of Mendelian randomization (Figure S2) (47, 53, 54). Therefore, to minimize the influence of pleiotropy, we have tried to remove pleiotropic instruments. In addition, we also carried out sensitivity analyses by excluding instruments that may be associated with other 38 complex phenotypes which may be associated with ALS in a metabolic, anthropometric, or socioeconomic way and possibly mediate the effect of BMI on ALS. Our Mendelian randomization analysis showed that the results are robust against pleiotropy and against various model assumptions.

### Possible Mechanisms Underlying the Associations Between BMI and ALS

The mechanisms underlying the observed associations between BMI and ALS is complex. Several explanations for such association exist. First, in observational studies it cannot fully remove the influence of measurement errors and confounding factors (e.g., cigarette smoking, alcohol drinking, or daily diet intakes) which can bias the observed association between BMI and ALS. Indeed, it was showed that BMI was no longer associated with ALS after controlling for socioeconomic status, prior chronic obstructive pulmonary disease, marital status, diabetes, and residence at ALS diagnosis (75). Second, it cannot completely rule out the possibility of reverse causality between BMI and ALS although it was not significant in our Mendelian randomization analysis. For example, previous studies showed that BMI may already begin to change before the onset of ALS (19), implying that the change of BMI may be the consequence rather than a risk factor of ALS. Finally, it cannot fully exclude the possible indirect effects of BMI on ALS although no direct effect was found in our Mendelian randomization analysis. For example, it is well-known that BMI is related to type II diabetes (T2D) (76) which in turn was showed to be associated with ALS (77), suggesting that there may exist an indirect influence of BMI on ALS via the pathway of T2D. Overall, further investigations are warranted to elaborate the relationship between BMI and ALS.

### Limitations of our Study

Some limitations of this study should be considered. First, similar to other Mendelian randomization studies, we acknowledge that the validity of our Mendelian randomization relies on three crucial modeling assumptions (Figure S2) (47, 53, 54). Although the first one (i.e., the relevant assumption) can be directly validated by examining the significance of SNPs on BMI, the second two assumptions are difficult to validate in practice. Thus, we emphasize that the results obtained in the present study should be explained cautiously, although we have implemented a lot of sensitivity analyses to guard against the misspecification of model assumptions. Second, also like other Mendelian randomization studies, we assumed a linear relationship between BMI and ALS in the Mendelian randomization model; while linearity may be not appropriate in the practice. Thus, we cannot fully exclude the possibility of non-linear association between BMI and ALS. Third, due to the fact that we conducted our analyses based on summary statistics rather than individual-level data sets, we cannot further investigate the causal effect between BMI and ALS in terms of gender or age (19, 34).

## Conclusion

In conclusion, based the Mendelian randomization results obtained from large-scale GWAS summary statistics, the present study is not supportive of the causal role of genetically increased or decreased BMI on the risk of ALS.

## Author Contributions

PZ and HX conceived the idea for the study. PZ and XY obtained the data and performed the data analyses. PZ interpreted the results of the data analyses. All the authors wrote and revised the manuscript.

### Conflict of Interest Statement

The authors declare that the research was conducted in the absence of any commercial or financial relationships that could be construed as a potential conflict of interest.
