# Supplementary material for: Association Between Premorbid Body Mass Index and Amyotrophic Lateral Sclerosis: Causal Inference Through Genetic Approaches
Source: Front Neurol. 2019 May 24;10:543. doi: 10.3389/fneur.2019.00543 (PMC6543002; doi:10.3389/fneur.2019.00543)
Supplement: Supplementary file 1 [file Data_Sheet_1.zip › 2019-04-09 Supplementary File S1.doc]

**Supplementary Text S1**

## Systematic Review and Meta-Analysis

### Data Sources and Search Strategies

Following the PRISMA guidelines , we carried out a literature search mainly on the database PubMed from January 2000 to September 2018 for articles on premorbid BMI (and related factors including overweight and obesity) and the risk of ALS. We also searched the articles about premorbid BMI and fronto temporal dementia (FTD) because of the shared pathological and genetic mechanism between ALS and FTD . Specifically, the search strategy included the following terms: "body mass index" or "BMI" or “premorbid body mass index” or “premorbid BMI” or “prediagnostic body mass index” or “prediagnostic BMI” or “presymptomatic body mass index” or “presymptomatic BMI” or “overweight” or “obesity”) and (“neurodegeneration” or “neurodegenerative diseases” or “neurodegenerative disorders” or “fronto temporal dementia” or “FTD” or “amyotrophic lateral sclerosis” or “ALS” or “motor neuron disease” or “Charcot disease” or “Lou Gehrig disease” or "frontotemporal lobe dementia") not ("animal") ("2000/01/01"[Date - Publication]: "2018/09/30"[Date - Publication]. We made no restriction on study designs and considered both cohort and population-based studies.

A total of 1,145 articles (1,134 articles by searching and additional 11 articles by references scanning) were obtained. Papers were excluded: (**i**) if the title and abstract clearly did not contain data on BMI and ALS; (**ii**) if there were insufficient results on BMI and ALS; (**iii**) duplicated papers and studies; (**iv**) if the paper was a review, letter-to-editor, response or commentary article; (**v**) if the paper explicitly showed a relationship between current (i.e. after-onset) BMI and ALS; and (**vi**) if it was a clinical drug trial. After filtering, 34 articles were kept for full-text reading, and 11 studies were finally included in the main systematic review and meta-analysis (FIGURE M1). All the studies were performed on individuals of European ancestry.

### Data Extraction

For each study, two investigators (PZ and XY) independently performed data extraction and quality assessment. We extracted from the article with the information on study setting, study design, population and case/control, observational period, measurement results of effect size [odds ratio (OR), relative ratio (RR) or hazard ratio (HR)] as well as possible covariates that were adjusted for in the analysis.

### Statistical analysis

The association between premorbid BMI and the risk of ALS was evaluated via inverse-variance weighted methods (both fixed and random effects meta-analyses were performed) by pooling all studies included in our analysis. The effect size heterogeneity among studies was tested by the Cochran’s Q statistic and was quantified with I^2^ . Published bias was assessed by the Egger method and funnel plot . Sensitivity analyses including the subgroup and leave-one-out analyses were also carried out to assess the robustness of the results.

**68** abstracts removed:

**56** no ALS/BMI

**3** review articles

**8** not reported BMI

**1** article not found

Titles and abstracts screened for **1,145** articles by PubMed MEDLINE

**1,134** articles identified by PubMed MEDLINE

**34** full-text articles read

**11** articles identified by citation screening

**102** abstracts of potentially relevant articles reviewed

**23** articles removed:

**4** not related to ALS

**16** about BMI after onset

**3** not included BMI

**11** studies included in final meta-analysis

FIGURE M1. Flowchart of the study selection process in the meta-analysis for investigating the association between premorbid BMI and the risk of ALS.

FIGURE M2. Combined OR of meta-analysis with the study of *Mariosa 2017a* removed. The study of *Mariosa 2017a* is case-control designed and reported the results for individuals when they aged at 25 years.

FIGURE M3. Combined OR of meta-analysis with the study of *Mariosa 2017b* removed. The study of *Mariosa 2017b* is case-control designed and reported the results for individuals when they aged at 45 years.

FIGURE M4. Combined OR of meta-analysis with the studies of *Doyle 2012* and *Aberg 2018* removed. The study of *Doyle 2012* focused only on women, while the study of *Aberg 2018* focused only on men. The other studies included in the analysis were performed on both sexes.

FIGURE M5. Combined OR of meta-analysis with only five prospective cohort studies included.

FIGURE M6. Combined OR of meta-analysis with only seven case-control studies included.

FIGURE M7. Combined OR of meta-analysis with only studies for which the main objective was to investigate the relationship between premorbid conditions (including premorbid BMI, weight or fat) and ALS.

FIGURE M8. Funnel plot displays the effect estimate for individual study of premorbid BMI on ALS.

Table M1

Leave-one-out analysis shows no single studies can substantially dominate the final pooled estimate in the meta-analysis.

| Removed | study | Fixed-effects model | | | | |  | Random-effects model | | | | |
| --- | --- | --- | --- | --- | --- | --- | --- | --- | --- | --- | --- | --- |
| study | year | OR | *L* | *U* | *z* | *p* |  | OR | *L* | *U* | *z* | *p* |
| Nelson | 2000 | 0.97 | 0.96 | 0.98 | -7.47 | 7.94E-14 |  | 0.97 | 0.95 | 0.98 | -5.48 | 4.14E-08 |
| Scarmeas | 2002 | 0.97 | 0.96 | 0.98 | -7.02 | 2.22E-12 |  | 0.97 | 0.96 | 0.98 | -4.75 | 2.01E-06 |
| Veldink | 2005 | 0.97 | 0.96 | 0.98 | -7.56 | 4.13E-14 |  | 0.97 | 0.95 | 0.98 | -5.67 | 1.42E-08 |
| Sutedja | 2011 | 0.97 | 0.96 | 0.98 | -7.00 | 2.58E-12 |  | 0.97 | 0.96 | 0.98 | -4.98 | 6.26E-07 |
| Doyle | 2012 | 0.96 | 0.95 | 0.97 | -7.05 | 1.73E-12 |  | 0.96 | 0.95 | 0.98 | -4.96 | 7.00E-07 |
| Gallo | 2013 | 0.97 | 0.96 | 0.98 | -7.17 | 7.38E-13 |  | 0.97 | 0.95 | 0.98 | -4.71 | 2.44E-06 |
| OReilly | 2013 | 0.97 | 0.96 | 0.98 | -6.26 | 3.85E-10 |  | 0.97 | 0.96 | 0.98 | -4.69 | 2.75E-06 |
| Huisman | 2015 | 0.97 | 0.96 | 0.98 | -6.99 | 2.70E-12 |  | 0.97 | 0.95 | 0.98 | -4.57 | 4.98E-06 |
| Mariosa | 2017a | 0.97 | 0.96 | 0.98 | -7.42 | 1.20E-13 |  | 0.97 | 0.95 | 0.98 | -5.10 | 3.37E-07 |
| Mariosa | 2017b | 0.97 | 0.96 | 0.98 | -6.94 | 3.86E-12 |  | 0.97 | 0.96 | 0.98 | -4.63 | 3.69E-06 |
| Aberg | 2018 | 0.97 | 0.96 | 0.98 | -6.93 | 4.08E-12 |  | 0.97 | 0.95 | 0.98 | -4.56 | 5.18E-06 |
| OReilly | 2018 | 0.97 | 0.96 | 0.98 | -6.84 | 7.93E-12 |  | 0.97 | 0.95 | 0.98 | -4.47 | 7.79E-06 |

Note: *L* and *U* are respectively the lower and upper for the 95% confidence interval of OR; *z* and *p* are respectively the *z* and *p* values for OR.

## References

1. Diekstra, F.P., Deerlin, V.M., Swieten, J.C., Al‐Chalabi, A., Ludolph, A.C., Weishaupt, J.H., et al. (2014). C9orf72 and UNC13A are shared risk loci for amyotrophic lateral sclerosis and frontotemporal dementia: A genome‐wide meta‐analysis. *Annals of Neurology* 76(1)**,** 120-133.
2. Egger, M., Davey Smith, G., Schneider, M., and Minder, C. (1997). Bias in meta-analysis detected by a simple, graphical test. *British Medical Journal* 315**,** 629-634.
3. Karch, C.M., Wen, N., Fan, C.C., and et al. (2018). Selective genetic overlap between amyotrophic lateral sclerosis and diseases of the frontotemporal dementia spectrum. *JAMA Neurology*. doi: 10.1001/jamaneurol.2018.0372.
4. Lattante, S., Ciura, S., Rouleau, G.A., and Kabashi, E. (2015). Defining the genetic connection linking amyotrophic lateral sclerosis (ALS) with frontotemporal dementia (FTD). *Trends in Genetics* 31(5)**,** 263-273. doi: 10.1016/j.tig.2015.03.005.
5. Moher, D., Liberati, A., Tetzlaff, J., and Altman, D.G. (2009). Preferred reporting items for systematic reviews and meta-analyses: the PRISMA statement. *BMJ (Clinical Research Ed.)* 339. doi: 10.1136/bmj.b2535.
6. Thompson, S.G., and Sharp, S.J. (1999). Explaining heterogeneity in meta-analysis: A comparison of methods. *Statistics in Medicine* 18(20)**,** 2693-2708. doi: 10.1002/(sici)1097-0258(19991030)18:20<2693::aid-sim235>3.0.co;2-v.
