# Supplementary material for: Association Between Premorbid Body Mass Index and Amyotrophic Lateral Sclerosis: Causal Inference Through Genetic Approaches
Source: Front Neurol. 2019 May 24;10:543. doi: 10.3389/fneur.2019.00543 (PMC6543002; doi:10.3389/fneur.2019.00543)
Supplement: Supplementary file 1 [file Data_Sheet_1.zip › 2019-04-09 Supplementary File S2.doc]

**Supplementary Text S2**

## Mendelian Randomization Analysis

# Model assumption of Mendelian randomization

In the literature of casual inference, Mendelian randomization is a special instrumental variable statistical method that is widely employed to evaluate the causal relationship between an exposure (i.e. body mass index (BMI)) and an outcome (i.e. amyotrophic lateral sclerosis (ALS)) in observational studies where the traditional randomized controlled trials are difficult to conduct . Briefly, Mendelian randomization uses genetic variants (e.g. single nucleotide polymorphisms (SNPs)) to serve as instrumental variables and estimates the causal effect of exposure on outcome . Intuitively, in the Mendelian randomization analysis we are dependent on the idea that SNPs which influence the exposure (e.g. BMI) would also affect the risk of outcome through the change of exposure (e.g. ALS).

Particularly, in Mendelian randomization each of the selected SNPs must meet three model assumptions (FIGURE 2): (**1**) the relevance assumption: the selected SNP is strongly associated with the exposure; (**2**) the independence assumption: the selected SNP is not associated with any other confounding factors that are potentially related to both the exposure and outcome; (**3**) the exclusion restriction assumption: the selected SNP has a direct influence on the outcome by the path of exposure alone. The recent successes of large-scale genome-wide association studies (GWASs) make it feasible to choose strongly associated SNPs to be valid instruments for causal inference in Mendelian randomization .

Note that, while the relevance assumption (the first one) can be directly validated by examining the significance of SNPs on the exposure, the second two assumptions (i.e. the independence and exclusion restriction assumptions) are difficult to validate in practice. Here we followed previous Mendelian randomization studies and performed a lot of sensitivity analyses to guard against these model assumptions.

## Estimation of causal effect in Mendelian randomization

We now describe details of the causal effect estimation in the Mendelian randomization analysis. Denote *G_i_* to be the genotype for a single instrumental variable *i* (i.e. a well-selected index SNP from large scale GWAS) (*i* = 1, 2, …, *m*, with *m* the number of instruments); and denote **x** to be the exposure and **y** be the outcome. Additionally, we assume all the model assumptions of instrumental variable mentioned above are satisfied (indicating *G_i_* is a valid instrument) and that *G_i_*, x and y are standardized so that they have mean zero and variance one. We employ the following linear model to characterize the relationship between *G_i_*, x and y

$$\begin{matrix} \text{x} = G_{i}\beta_{i}^{x} + e^{x}, e^{x} \sim N(0, \text{σ}_{gx}^{2}), \\ \text{y} = \text{x}\theta+ \varepsilon^{xy}, \varepsilon^{xy} \sim N(0, \text{σ}_{y}^{2}), \end{matrix}$$

where the intercept term is not included in model (1) since those variables are previously assumed to be standardized; $\beta_{i}^{x}$ is the effect sizes of instrumental variable *i* for the exposure and outcome, respectively; and *θ_i_* is the causal effect of x on y; $e^{x}$ and $\varepsilon^{xy}$ are assumed to be normal residuals with variances of $\text{σ}_{gx}^{2}$ and $\text{σ}_{y}^{2}$. Then, *θ_i_* can be estimated by the two-stage least squares method.

However, the application of model requires the individual-level data for the instrument as well as for the exposure and outcome, which are not always available. Mendelian randomization methods which evaluate the causal effect using only summary-level data are recently developed (referred as to two-sample Mendelian randomization). Let $\hat{\beta}_{i}^{x}$ and $\text{var}(\hat{\beta}_{i}^{x})$ be the effect size and variance of instrumental variable *i* for the exposure, respectively, and let the effect size and variance for the same instrumental variable *i* on y be $\hat{\beta}_{i}^{y}$ and $\text{var}(\hat{\beta}_{i}^{y})$, respectively. We can estimate the causal effect *θ_i_* of the exposure on the outcome simply by

$\hat{\theta}_{i} = \hat{\beta}_{i}^{y}/\hat{\beta}_{i}^{x}$.

Furthermore, supposing both $\hat{\beta}_{i}^{x}$ and $\hat{\beta}_{i}^{y}$ follow a normal distribution we can estimate the asymptotic variance of $\hat{\theta}_{i}$ with the delta method

$\text{var}(\hat{\theta}_{i}) \approx\frac{\text{var}(\hat{\beta}_{i}^{y})}{(\hat{\beta}_{i}^{x})^{2}} + \frac{(\hat{\beta}_{i}^{y})^{2}\text{var}(\hat{\beta}_{i}^{x})}{(\hat{\beta}_{i}^{x})^{4}}$.

When there are multiple independent instrumental variables, we combine the above estimates from all the instrumental variables to generate a pooled estimate of the causal effect through the inverse-variance weighted method

$\hat{\theta} = \frac{1}{\sum_{i=1}^{m} \text{var}(\hat{\beta}_{i}^{y})^{-1}(\hat{\beta}_{i}^{x})^{2}}\sum_{i=1}^{m} \text{var}(\hat{\beta}_{i}^{y})^{-1}\hat{\beta}_{i}^{y}\hat{\beta}_{i}^{x}$,

with the variance as

$\text{Var}(\hat{\theta}) = \frac{1}{\sum_{i=1}^{m} \text{var}(\hat{\beta}_{i}^{y})^{-1}(\hat{\beta}_{i}^{x})^{2}}$.

# GWAS Data Sources

BMI data for the European population

We employed a large-scale BMI GWAS data set for the European population in our analyses. The data set was created by the Genetic Investigation of ANthropometric Traits (GIANT) consortium, and is the largest BMI GWAS that was formally published and publicly available to date . The GIANT BMI study is a meta-analysis that combined individuals of European ancestry from the UK BioBank and subjects from study of Locke et al .

In the study of Locke et al , 2,554,637 genotyped and imputed SNPs were meta-analyzed up on 322,154 individuals of European ancestry from 125 sub-studies. BMI was first adjusted for age, age^2^ and study-specific covariates in a linear regression model and genetic relatedness was also adjusted for in a study-specific manner. The resulting residuals were finally quintile normalized to a standard normal distribution before association analysis.

In the UK BioBank, 16,652,994 genotyped and imputed SNPs up on 451,099 individuals of European ancestry were analyzed. BMI was first adjusted for gender, age, ten principal components, recruitment center and genotyping batches, and then the resulting residuals were finally quintile normalized to a standard normal distribution before association analysis. The association was analyzed with an efficient Bayesian linear mixed model to further account for genetic relatedness between participants and population stratification .

Finally, the UK BioBank results and the results in Locke et al were pooled together by the fixed-effect inverse-variance weighted meta-analysis method up on a total of ~770,000 individuals and 2,336,269 shared SNPs ; the SNPs association was further performed using the conditional and joint analysis (COJO) . The summary results of BMI can be publicly available from <http://portals.broadinstitute.org/collaboration/giant/index.php/Main_Page>.

BMI data for the East Asian population

As a further validation analysis, we also selected index SNPs to serve as instruments for BMI in the East Asian population. This East Asian BMI GWAS analyzed a total of 6,108,953 genotyped and imputed SNPs up on 158,284 Japanese individuals . BMI was first controlled for the effects of gender, age, age^2^, top ten principal components; and then the resulting residuals were quintile normalized to a standard normal distribution before association analysis. The summary results can be publicly available from http://jenger.riken.jp/en/.

ALS data for the European population

The ALS GWAS used in the present study is the largest ALS Meta GWAS to date in the European population and was a case-control study that analyzed 10,031,417 genotyped and imputed SNPs up on 80,610 individuals (20,806 cases and 59,804 controls) of European ancestry . Logistic regression was performed for each sub-study with principal components, age and gender as covariates. The association analysis was finally implemented using the fixed-effect inverse-variance weighted method across sub-studies. The summary results can be publicly available from https://www.ebi.ac.uk/gwas/home.

ALS data for the East Asian population

The East Asian ALS GWAS was performed on 4,084 Chinese individuals (1,234 cases and 2,850 controls) for 6,613,544 genotyped and imputed SNP .

For all the four GWASs, we obtained association summary statistic for each SNPs available in terms of the effect allele, marginal effect size estimate for BMI or log odds ratio (OR) for ALS and standard error.

# Select instruments for BMI

We first selected index SNPs to serve as instrumental variables for BMI in the European population. As no independent index association loci were directly available in Yengo et al , within the GIANT study we carefully generated a set of independent index SNPs that are associated with BMI at the genome-wide significance level by applying the clumping procedure of PLINK (version v1.90b3.38) . Following , we set both the significance threshold and the secondary significance threshold for index SNPs to be 5.00E-8, and set the linkage disequilibrium (LD) threshold and the physical distance threshold to be 0.01 and 1,000, respectively. The LD was estimated using a reference panel of 503 individuals of European ancestry in the 1000 Genomes Project .

After doing this, we created a set of 1,031 independent index association SNPs (*p*<5.00E-8) as instrumental variables (Table S3). Note that, rather than the commonly-used level of 5.00E-8 in GWAS, in the COJO association analysis a more conservative significance level of 1.00E-8 is typically recommended , which resulted in a total of 941 independent index association SNPs in the original study of . However, the list of those SNPs is not available. Using the COJO significance level of 1.00E-8 we obtained 887 independent index association SNPs (all of them were included in the previous set of instruments). Based on those 887 index SNPs we generated a similar null causal effect estimate for BMI on ALS (OR=1.03, 95% CI 0.96~1.10, *p*=0.392) using the IVW method. As the power of Mendelian randomization analysis typically increases as the number of instruments and the significance level of 5.00E-8 is still valid for this BMI GWAS , we thus applied 1,031 instruments in our main analysis.

We directly obtained a set of 75 index association SNPs (*p*<5.00E-8) to serve as instrumental variables for BMI from the East Asian GWAS (Table S4) .

For the instruments of BMI from the GIANT study , we extracted corresponding association summary statistics for ALS from the GWAS of European ancestry in the form of effect allele, log OR and standard error; while for these instruments of BMI from the East Asian study , we extracted corresponding association summary statistics for ALS from the GWAS of East Asian ancestry . In both the populations, all the selected instruments for BMI can be available in the respective ALS GWAS summary result.

Exclude reverse causality between BMI and ALS

We carried out a reverse causal inference to formally exclude the possibility that the causal relationship between BMI and ALS was due to a causal effect of ALS on BMI. To do so, we performed a Mendelian randomization analysis in the reverse direction by using ALS associated SNPs as instrumental variables. In the European population, we obtained a total of 6 ALS associated SNPs (*p*<5.00E-8) to serve as instruments . However, for these SNP instruments, only one (rs3849943) can be available in the BMI GIANT GWAS data set .

# Other 38 complex metabolic, anthropometric and socioeconomic traits from large scale GWASs

Due to the potential relationship between ALS patients and energy metabolism , as a sensitivity analysis to guard against the possible pleiotropic effects, we further removed instruments that may be associated with other 38 complex metabolic, anthropometric and socioeconomic traits from large scale GWASs for which summary statistics results can be publicly available.

A total of 24 metabolic traits were included: adiponectin level , glycated hemoglobin (HbA1c) , insulin sensitivity index (ISI) , glucose level 2 hours after an oral glucose challenge (2-h glucose) , fasting glucose, fasting insulin and indices of *β*-cell function (HOMA-B) and insulin resistance (HOMA-IR) , acute insulin response, disposition index, insulin secretion rate and peak insulin response , leptin level , high density lipoprotein (HDL), low density lipoprotein (LDL), total cholesterol (TC) and triglycerides (TG) , type 2 diabetes (T2D) , coronary artery disease (CAD) , diastolic blood pressure (DBP) and systolic blood pressure (SBP) , heart hate , C-reactive protein and uric acid .

A total of 7 anthropometric traits were included: body fat percentage , obesity and overweight , weight, waist circumference, waist-hip ratio and hip circumference .

A total of 7 socioeconomic traits were included: cigarettes per day, ever smoking, former smoker and age onset of smoking , education years , college and household income .

For all the complex traits above, we obtained association summary statistic for each SNPs in terms of the effect allele, marginal effect size estimate or log odds ratio (OR) and standard error from the public portal or individual level data.

**FIGURE S1** Body mass index (BMI) for cases and controls before ALS onset in the study of Peter et al (2017)

**FIGURE S2** Graphical framework for illustrating Mendelian randomization. Solid arrows or dotted arrows represent the presence or absence of associations. Valid Mendelian randomization analysis requires three critical modeling assumptions. Assumption I: the instruments are strongly associated with the exposure (i.e. BMI). Assumption II: the instruments are not correlated to the any confounders of BMI and ALS. Assumption III: the instruments are independent of ALS conditional on BMI. SNP: single nucleotide polymorphisms; BMI: body mass index; ALS: amyotrophic lateral sclerosis.

**Table S1 Summary of the effect sizes for the increasing premorbid BMI and ALS in previous studies.**

| Note | OR/Mean (SD) | 95% Confidence  Interval | |  | Ref |
| --- | --- | --- | --- | --- | --- |
|  |  | Lower | Upper |  |  |
| **BMI** |  |  |  |  |  |
| Normal | 1.00 | 1.00 | 1.00 |  |  |
| Underweight | 2.79 | 1.35 | 5.77 |  |  |
| Overweight | 0.83 | 0.61 | 1.14 |  |  |
| Obesity | 0.92 | 0.60 | 1.40 |  |  |
| **BMI** (men) |  |  |  |  |  |
| Normal | 1.00 | 1.00 | 1.00 |  |  |
| Underweight | - |  |  |  |  |
| Overweight | 0.78 | 0.48 | 1.27 |  |  |
| Obesity | 0.44 | 0.19 | 1.02 |  |  |
| Continuous | 0.93 | 0.86 | 0.99 |  |  |
| **BMI** (women) |  |  |  |  |  |
| Normal | 1.00 | 1.00 | 1.00 |  |  |
| Underweight | 3.36 | 1.61 | 7.04 |  |  |
| Overweight | 0.81 | 0.54 | 1.21 |  |  |
| Obesity | 1.24 | 0.75 | 2.04 |  |  |
| Continuous | 0.99 | 0.95 | 1.03 |  |  |
| **BMI** |  |  |  |  |  |
| First quartile | 1.00 | 1.00 | 1.00 |  |  |
| Second quartile | 0.76 | 0.52 | 1.10 |  |  |
| Third quartile | 0.62 | 0.42 | 0.92 |  |  |
| Fourth quartile | 0.70 | 0.47 | 1.04 |  |  |
| Continuous | 0.97 | 0.94 | 1.01 |  |  |
| **Sensitivity analysis** |  |  |  |  |  |
| **BMI** (Men) |  |  |  |  |  |
| Underweight | - |  |  |  |  |
| Normal | 1.00 | 1.00 | 1.00 |  |  |
| Overweight | 0.87 | 0.52 | 1.46 |  |  |
| Obesity | 0.47 | 0.19 | 1.18 |  |  |
| Continuous | 0.94 | 0.87 | 1.01 |  |  |
| **BMI** (Women) |  |  |  |  |  |
| Normal | 1.00 | 1.00 | 1.00 |  |  |
| Underweight | 3.74 | 1.78 | 7.87 |  |  |
| Overweight | 0.84 | 0.55 | 1.28 |  |  |
| Obesity | 1.30 | 0.77 | 2.19 |  |  |
| Continuous | 0.99 | 0.95 | 1.04 |  |  |
| **BMI** |  |  |  |  |  |
| First quartile | 1.00 | 1.00 | 1.00 |  |  |
| Second quartile | 0.84 | 0.57 | 1.25 |  |  |
| Third quartile | 0.67 | 0.45 | 1.02 |  |  |
| Fourth quartile | 0.78 | 0.51 | 1.18 |  |  |
|  |  |  |  |  |  |
| **BMI** (Netherlands) | 0.92 | 0.87 | 0.97 |  |  |
| **BMI** (USA; 5 years before onset) |  |  |  |  |  |
| 21-24 vs <21 | 1.70 | 0.80 | 3.60 |  |  |
| 24-26 vs <21 | 1.50 | 0.70 | 3.30 |  |  |
| >26 vs <21 | 1.70 | 0.80 | 3.70 |  |  |
|  |  |  |  |  |  |
| **BMI** (USA) |  |  |  |  |  |
| Obesity (BMI>30 vs BMI<25) | 0.71 | 0.31 | 1.61 |  |  |
| Overweight (25<BMI<30 vs BMI<25) | 0.88 | 0.35 | 2.67 |  |  |
| Continuous | 0.94 | 0.90 | 0.99 |  |  |
|  |  |  |  |  |  |
| **BMI** (14-28-year follow-up) |  |  |  |  |  |
| BMI | 0.95 | 0.94 | 0.97 |  |  |
| overweight | 0.95 | 0.91 | 0.99 |  |  |
| obesity | 0.94 | 0.89 | 0.99 |  |  |
| **All individuals** |  |  |  |  |  |
| <18.5 vs 18.5-23 | 1.61 | 0.88 | 2.94 |  |  |
| 23-25 vs 18.5-23 | 0.80 | 0.67 | 0.95 |  |  |
| 25-27.5 vs 18.5-23 | 0.71 | 0.59 | 0.85 |  |  |
| 2.75-30 vs 18.5-23 | 0.62 | 0.49 | 0.78 |  |  |
| >30 vs 18.5-23 | 0.63 | 0.49 | 0.81 |  |  |
| **Men** |  |  |  |  |  |
| <18.5 vs 18.5-23 | 2.52 | 0.34 | 18.5 |  |  |
| 23-25 vs 18.5-23 | 0.68 | 0.53 | 0.86 |  |  |
| 25-27.5 vs 18.5-23 | 0.64 | 0.50 | 0.83 |  |  |
| 2.75-30 vs 18.5-23 | 0.59 | 0.45 | 0.78 |  |  |
| >30 vs 18.5-23 | 0.58 | 0.36 | 0.94 |  |  |
| **Women** |  |  |  |  |  |
| <18.5 vs 18.5-23 | 1.25 | 0.70 | 2.26 |  |  |
| 23-25 vs 18.5-23 | 0.96 | 0.75 | 1.25 |  |  |
| 25-27.5 vs 18.5-23 | 0.79 | 0.61 | 1.04 |  |  |
| 2.75-30 vs 18.5-23 | 0.64 | 0.41 | 0.99 |  |  |
| >30 vs 18.5-23 | 0.68 | 0.51 | 0.92 |  |  |
| **No smoking** |  |  |  |  |  |
| <18.5 vs 18.5-23 | 2.74 | 1.39 | 5.41 |  |  |
| 23-25 vs 18.5-23 | 0.85 | 0.63 | 1.14 |  |  |
| 25-27.5 vs 18.5-23 | 0.71 | 0.53 | 0.96 |  |  |
| 2.75-30 vs 18.5-23 | 0.64 | 0.48 | 1.00 |  |  |
| >30 vs 18.5-23 | 0.59 | 0.41 | 0.86 |  |  |
| **Study-specific estimates** |  |  |  |  |  |
| NIH-AARP (Women) | 0.73 | 0.55 | 0.97 |  |  |
| CPS II-N (Women) | 0.84 | 0.61 | 1.16 |  |  |
| MEC (Women) | 0.70 | 0.45 | 1.08 |  |  |
| NHS 1980-1994 | 0.96 | 0.52 | 1.76 |  |  |
| NHS 1994-2008 | 0.85 | 0.60 | 1.22 |  |  |
| NIH-AARP (Men) | 0.67 | 0.50 | 0.89 |  |  |
| CPS II-N (Men) | 0.80 | 0.49 | 1.32 |  |  |
| MEC (Men) | 0.47 | 0.24 | 0.92 |  |  |
| HPFS | 0.70 | 0.33 | 1.45 |  |  |
| Overall | 0.75 | 0.66 | 0.85 |  |  |
| **Excluded the first 4/7 years** |  |  |  |  |  |
| Four years | 0.80 | 0.72 | 0.89 |  |  |
| Seven years | 0.82 | 0.72 | 0.93 |  |  |
|  |  |  |  |  |  |
| **BMI** (Netherlands) | 0.97 | 0.94 | 0.99 |  |  |
|  |  |  |  |  |  |
| **BMI** |  |  |  |  |  |
| BMI at age 25 years | 0.99 | 0.95 | 1.03 |  |  |
| Category of BMI at age 25 years |  |  |  |  |  |
| 15–<20 vs 20–<25 | 0.96 | 0.63 | 1.46 |  |  |
| 25–<30 vs 20–<25 | 1.08 | 0.83 | 1.39 |  |  |
| ≥30.0 vs 20–<25 | 0.76 | 0.37 | 1.45 |  |  |
|  |  |  |  |  |  |
| BMI at age 40 years | 0.95 | 0.91 | 0.98 |  |  |
| Category of BMI at age 40 years |  |  |  |  |  |
| 15–<20 vs 20–<25 | 0.95 | 0.40 | 2.26 |  |  |
| 25–<30 vs 20–<25 | 0.84 | 0.65 | 1.08 |  |  |
| ≥30.0 vs 20–<25 | 0.59 | 0.39 | 0.90 |  |  |
|  |  |  |  |  |  |
| BMI at reference date | 0.90 | 0.88 | 0.93 |  |  |
| Category of BMI at reference date |  |  |  |  |  |
| 15–<20 vs 20–<25 | 2.27 | 0.90 | 5.71 |  |  |
| 25–<30 vs 20–<25 | 0.63 | 0.48 | 0.83 |  |  |
| ≥30.0 vs 20–<25 | 0.34 | 0.24 | 0.47 |  |  |
|  |  |  |  |  |  |
| BMI at age 25 years | 0.99 | 0.95 | 1.03 |  |  |
| Category of BMI at age 25 years |  |  |  |  |  |
| 15–<20 vs 20–<25 | 0.88 | 0.57 | 1.35 |  |  |
| 25–<30 vs 20–<25 | 1.07 | 0.82 | 1.38 |  |  |
| ≥30.0 vs 20–<25 | 0.71 | 0.36 | 1.42 |  |  |
|  |  |  |  |  |  |
| BMI at age 40 years | 0.95 | 0.91 | 0.98 |  |  |
| Category of BMI at age 40 years |  |  |  |  |  |
| 15–<20 vs 20–<25 | 0.92 | 0.38 | 2.22 |  |  |
| 25–<30 vs 20–<25 | 0.85 | 0.65 | 1.10 |  |  |
| ≥30.0 vs 20–<25 | 0.60 | 0.39 | 0.92 |  |  |
|  |  |  |  |  |  |
| BMI at reference date | 0.90 | 0.88 | 0.93 |  |  |
| Category of BMI at reference date |  |  |  |  |  |
| 15–<20 vs 20–<25 | 2.21 | 0.87 | 5.66 |  |  |
| 25–<30 vs 20–<25 | 0.66 | 0.50 | 0.87 |  |  |
| ≥30.0 vs 20–<25 | 0.35 | 0.25 | 0.49 |  |  |
|  |  |  |  |  |  |
| **BMI Between Ages 25 and 40 Years** |  |  |  |  |  |
| BMI change | 0.94 | 0.89 | 1.00 |  |  |
| Category of change in BMI |  |  |  |  |  |
| Decrease or stable | 1.61 | 1.21 | 2.14 |  |  |
| Substantial increase | 1.06 | 0.77 | 1.45 |  |  |
| BMI change | 0.94 | 0.89 | 1.00 |  |  |
| Category of change in BMI |  |  |  |  |  |
| Decrease or stable | 1.61 | 1.20 | 2.16 |  |  |
| Substantial increase | 1.06 | 0.76 | 1.47 |  |  |
| **BMI Between at 40 Years** |  |  |  |  |  |
| BMI at age 40 years | 0.95 | 0.92 | 0.99 |  |  |
| 15–<20 vs 20–<25 | 1.02 | 0.41 | 2.55 |  |  |
| 25–<30 vs 20–<25 | 0.85 | 0.64 | 1.12 |  |  |
| ≥30.0 vs 20–<25 | 0.68 | 0.44 | 1.07 |  |  |
| BMI at age 40 years | 0.93 | 0.85 | 1.01 |  |  |
| 15–<20 vs 20–<25 | 0.76 | 0.10 | 6.05 |  |  |
| 25–<30 vs 20–<25 | 0.93 | 0.53 | 1.65 |  |  |
| ≥30.0 vs 20–<25 | 0.25 | 0.06 | 1.10 |  |  |
|  |  |  |  |  |  |
| **BMI Between at 40 Years (Delay)** |  |  |  |  |  |
| BMI at age 40 years | 0.91 | 0.87 | 0.96 |  |  |
| 15–<20 vs 20–<25 | 1.22 | 0.43 | 3.46 |  |  |
| 25–<30 vs 20–<25 | 0.72 | 0.51 | 1.02 |  |  |
| ≥30.0 vs 20–<25 | 0.44 | 0.23 | 0.81 |  |  |
| BMI at age 40 years | 0.96 | 0.91 | 1.02 |  |  |
| 15–<20 vs 20–<25 | 0.36 | 0.05 | 2.77 |  |  |
| 25–<30 vs 20–<25 | 0.89 | 0.60 | 1.32 |  |  |
| ≥30.0 vs 20–<25 | 0.78 | 0.41 | 1.45 |  |  |
| BMI at age 40 years | 1.01 | 0.95 | 1.09 |  |  |
| 15–<20 vs 20–<25 | 1.60 | 0.34 | 7.45 |  |  |
| 25–<30 vs 20–<25 | 1.38 | 0.80 | 2.38 |  |  |
| ≥30.0 vs 20–<25 | 0.92 | 0.38 | 2.25 |  |  |
|  |  |  |  |  |  |
| **BMI Between at 40 Years (Survival)** |  |  |  |  |  |
| BMI at age 40 years | 1.01 | 0.97 | 1.04 |  |  |
| 15–<20 vs 20–<25 | 1.39 | 0.61 | 3.19 |  |  |
| 25–<30 vs 20–<25 | 0.95 | 0.75 | 1.21 |  |  |
| ≥30.0 vs 20–<25 | 1.11 | 0.73 | 1.67 |  |  |
| BMI at age 40 years | 1.02 | 0.98 | 1.05 |  |  |
| 15–<20 vs 20–<25 | 1.36 | 0.60 | 3.12 |  |  |
| 25–<30 vs 20–<25 | 0.98 | 0.77 | 1.25 |  |  |
| ≥30.0 vs 20–<25 | 1.22 | 0.81 | 1.85 |  |  |
|  |  |  |  |  |  |
| **BMI Between at diagnosis (Survival)** |  |  |  |  |  |
| BMI at diagnosis | 0.97 | 0.94 | 1.00 |  |  |
| 15–<20 vs 20–<25 | 1.64 | 0.89 | 3.04 |  |  |
| 25–<30 vs 20–<25 | 0.87 | 0.68 | 1.11 |  |  |
| ≥30.0 vs 20–<25 | 0.70 | 0.50 | 0.97 |  |  |
| BMI at diagnosis | 0.98 | 0.95 | 1.01 |  |  |
| 15–<20 vs 20–<25 | 1.63 | 0.88 | 3.02 |  |  |
| 25–<30 vs 20–<25 | 0.90 | 0.70 | 1.17 |  |  |
| ≥30.0 vs 20–<25 | 0.76 | 0.54 | 1.07 |  |  |
|  |  |  |  |  |  |
| **Premorbid BMI** | 0.96 | 0.93 | 0.99 |  |  |
|  |  |  |  |  |  |
| **Premorbid BMI** | 0.97 | 0.95 | 0.99 |  |  |
| **Women** | 0.87 | 0.77 | 0.99 |  |  |
| BCDDP | 1.19 | 0.74 | 1.91 |  |  |
| CTS | 0.78 | 0.57 | 1.07 |  |  |
| IWHS | 0.74 | 0.57 | 0.96 |  |  |
| MCCS-W | 1.04 | 0.58 | 1.88 |  |  |
| NLCS-W | 0.80 | 0.49 | 1.30 |  |  |
| PLCO-W | 0.84 | 0.55 | 1.29 |  |  |
| SMC | 0.98 | 0.69 | 1.40 |  |  |
| WHS | 1.07 | 0.77 | 1.48 |  |  |
| WLHS | 0.68 | 0.32 | 1.43 |  |  |
| **Men** | 0.75 | 0.56 | 1.02 |  |  |
| COSM | 0.99 | 0.69 | 1.42 |  |  |
| MCCS-M | 0.41 | 0.18 | 0.91 |  |  |
| NLCS-M | 0.75 | 0.47 | 1.19 |  |  |
| PLCO-M | 0.68 | 0.41 | 1.13 |  |  |
|  |  |  |  |  |  |
| **Premorbid BMI** |  |  |  |  |  |
| **Main analysis** |  |  |  |  |  |
| <20.0 | 1.14 | 0.79 | 1.65 |  |  |
| 20.0~25.0 | 1.00 | 0.89 | 1.12 |  |  |
| 25.0~30.0 | 0.87 | 0.74 | 1.03 |  |  |
| 30.0~35.0 | 0.82 | 0.64 | 1.04 |  |  |
| >35.0 | 0.73 | 0.49 | 1.07 |  |  |
| **Excluding the first 3 years of follow-up** |  |  |  |  |  |
| <20.0 | 1.14 | 0.75 | 1.71 |  |  |
| 20.0~25.0 | 1.00 | 0.88 | 1.13 |  |  |
| 25.0~30.0 | 0.80 | 0.66 | 0.96 |  |  |
| 30.0~35.0 | 0.84 | 0.64 | 1.10 |  |  |
| >35.0 | 0.61 | 0.38 | 0.97 |  |  |
| **Premorbid BMI** | 1.02 | 0.97 | 1.08 |  |  |
| >30 vs <25 | 1.36 | 0.92 | 2.00 |  |  |
| 25~30 vs <25 | 0.95 | 0.47 | 1.89 |  |  |

**Table S2** BMI for cases and controls before ALS onset in the study of Peter et al (2017) .

| BMI (years before onset) | Case | |  | Control | |
| --- | --- | --- | --- | --- | --- |
|  | *n* | Mean (SD) |  | *n* | Mean (SD) |
| 55-65 | 67 | 22.80 (2.50) |  | 133 | 21.90 (2.70) |
| 45-55 | 186 | 23.00 (2.90) |  | 451 | 22.50 (2.90) |
| 35-45 | 282 | 23.50 (3.00) |  | 657 | 23.00 (3.00) |
| 25-35 | 337 | 24.30 (3.40) |  | 753 | 23.80 (3.20) |
| 15-25 | 357 | 25.30 (3.80) |  | 771 | 24.80 (3.60) |
| 5-15 | 314 | 26.20 (4.10) |  | 675 | 25.90 (4.20) |
| 1-5 | 74 | 26.10 (4.80) |  | 174 | 25.80 (3.90) |
| 0 | 393 | 24.60 (4.20) |  | 791 | 26.50 (4.10) |

**Table S3** 1,031 independent index association SNPs (*p*<5.00E-8) to serve as instrumental variables for BMI from the Genetic Investigation of ANthropometric Traits (GIANT) consortium (shown in an excel file).

**Table S4** 75 independent instruments obtained from an East Asian BMI GWAS (shown in an excel file).

**Table S5 Estimate of the causal effect of BMI on ALS after removing instruments that may be potentially associated with other 38 complex traits with various threshold values.**

| Threshold | *k* | OR (95% CI) | p value |
| --- | --- | --- | --- |
| 1.00E-8 | 970 | 1.03 (0.96~1.11) | 0.172 |
| 5.00E-8 | 959 | 1.03 (0.96~1.11) | 0.179 |
| 1.00E-7 | 955 | 1.04 (0.96~1.11) | 0.172 |
| 1.00E-6 | 923 | 1.04 (0.97~1.12) | 0.135 |
| 1.00E-5 | 870 | 1.03 (0.96~1.11) | 0.213 |
| 1.00E-4 | 756 | 1.03 (0.95~1.12) | 0.235 |
| 1.00E-3 | 543 | 1.03 (0.93~1.14) | 0.282 |
| 1.00E-2 | 201 | 1.01 (0.85~1.20) | 0.469 |

Note: *k* is the number of instruments used in the analysis after removing these that may be potentially associated with other 38 complex traits. Using all the instruments, the OR was estimated to be 1.04 (95% CI 0.97~1.11, *p*=0.275).

## References

1. Aberg, M., Nyberg, J., Robertson, J., Kuhn, G., Schioler, L., Nissbrandt, H., et al. (2018). Risk factors in Swedish young men for amyotrophic lateral sclerosis in adulthood. *Journal of Neurology* 265(3)**,** 460-470. doi: 10.1007/s00415-017-8719-1.
2. Akiyama, M., Okada, Y., Kanai, M., Takahashi, A., Momozawa, Y., Ikeda, M., et al. (2017). Genome-wide association study identifies 112 new loci for body mass index in the Japanese population. *Nature Genetics* 49(10)**,** 1458-1467. doi: 10.1038/ng.3951.
3. Angrist, J.D., Imbens, G.W., and Rubin, D.B. (1996). Identification of Causal Effects Using Instrumental Variables. *Journal of the American Statistical Association* 91(434)**,** 444-455. doi: 10.1080/01621459.1996.10476902.
4. Benyamin, B., He, J., Zhao, Q., Gratten, J., Garton, F., Leo, P.J., et al. (2017). Cross-ethnic meta-analysis identifies association of the GPX3-TNIP1 locus with amyotrophic lateral sclerosis. *Nature Communications* 8(1)**,** 611.
5. Berndt, S.I., Gustafsson, S., Magi, R., Ganna, A., Wheeler, E., Feitosa, M.F., et al. (2013). Genome-wide meta-analysis identifies 11 new loci for anthropometric traits and provides insights into genetic architecture. *Nature Genetics* 45(5)**,** 501-512. doi: 10.1038/ng.2606.
6. Burgess, S., Small, D.S., and Thompson, S.G. (2017). A review of instrumental variable estimators for Mendelian randomization. *Statistical Methods in Medical Research* 26(5)**,** 2333-2355. doi: 10.1177/0962280215597579
7. Burgess, S., and Thompson, S.G. (2017). Interpreting findings from Mendelian randomization using the MR-Egger method. *European Journal of Epidemiology* 32(5)**,** 377-389. doi: 10.1007/s10654-017-0255-x.
8. Dastani, Z., Hivert, M.-F., Timpson, N., Perry, J.R.B., Yuan, X., Scott, R.A., et al. (2012). Novel Loci for Adiponectin Levels and Their Influence on Type 2 Diabetes and Metabolic Traits: A Multi-Ethnic Meta-Analysis of 45,891 Individuals. *PLoS Genetics* 8(3)**,** e1002607. doi: 10.1371/journal.pgen.1002607.
9. den Hoed, M., Eijgelsheim, M., Esko, T., Brundel, B.J.J.M., Peal, D.S., Evans, D.M., et al. (2013). Identification of heart rate-associated loci and their effects on cardiac conduction and rhythm disorders. *Nature Genetics* 45(6)**,** 621-631. doi: 10.1038/ng.2610.
10. Doyle, P., Brown, A., Beral, V., Reeves, G., and Green, J. (2012). Incidence of and risk factors for Motor Neurone Disease in UK women: a prospective study. *BMC Neurology* 12(1)**,** 25. doi: 10.1186/1471-2377-12-25.
11. Dupuis, J., Langenberg, C., Prokopenko, I., Saxena, R., Soranzo, N., Jackson, A.U., et al. (2010). New genetic loci implicated in fasting glucose homeostasis and their impact on type 2 diabetes risk. *Nature Genetics* 42(2)**,** 105-116. doi: 10.1038/ng.520.
12. Dupuis, L., Pradat, P.-F., Ludolph, A.C., and Loeffler, J.-P. (2011). Energy metabolism in amyotrophic lateral sclerosis. *Lancet Neurology* 10(1)**,** 75-82. doi: https://doi.org/10.1016/S1474-4422(10)70224-6.
13. Gallo, V., Wark, P.A., Jenab, M., Pearce, N., Brayne, C., Vermeulen, R., et al. (2013). Prediagnostic body fat and risk of death from amyotrophic lateral sclerosis The EPIC cohort. *Neurology* 80(9)**,** 829-838. doi: 10.1212/WNL.0b013e3182840689.
14. Hartwig, F.P., Davey Smith, G., and Bowden, J. (2017). Robust inference in summary data Mendelian randomization via the zero modal pleiotropy assumption. *International Journal of Epidemiology* 46(6)**,** 1985-1998. doi: 10.1093/ije/dyx102
15. Hill, W D., Hagenaars, Saskia P., Marioni, Riccardo E., Harris, Sarah E., Liewald, David C., Davies, G., et al. (2016). Molecular Genetic Contributions to Social Deprivation and Household Income in UK Biobank. *Current Biology* 26(22)**,** 3083-3089. doi: 10.1016/j.cub.2016.09.035.
16. Huisman, M.H.B., Seelen, M., van Doormaal, P.T.C., de Jong, S.W., de Vries, J.H.M., van der Kooi, A.J., et al. (2015). Effect of Presymptomatic Body Mass Index and Consumption of Fat and Alcohol on Amyotrophic Lateral Sclerosis. *JAMA Neurology* 72(10)**,** 1155-1162. doi: 10.1001/jamaneurol.2015.1584.
17. Kilpeläinen, T.O., Carli, J.F.M., Skowronski, A.A., Sun, Q., Kriebel, J., Feitosa, M.F., et al. (2016). Genome-wide meta-analysis uncovers novel loci influencing circulating leptin levels. *Nature Communications* 7**,** 10494. doi: 10.1038/ncomms10494.
18. Lawlor, D.A., Harbord, R.M., Sterne, J.A., Timpson, N., and Davey Smith, G. (2008). Mendelian randomization: using genes as instruments for making causal inferences in epidemiology. *Statistics in Medicine* 27(8)**,** 1133-1163. doi: 10.1002/sim.3034.
19. Locke, A.E., Kahali, B., Berndt, S.I., Justice, A.E., Pers, T.H., Day, F.R., et al. (2015). Genetic studies of body mass index yield new insights for obesity biology. *Nature* 518(7538)**,** 197-206.
20. Loh, P.-R., Kichaev, G., Gazal, S., Schoech, A.P., and Price, A.L. (2018). Mixed-model association for biobank-scale datasets. *Nature Genetics* 50(7)**,** 906-908. doi: 10.1038/s41588-018-0144-6.
21. Loh, P.-R., Tucker, G., Bulik-Sullivan, B.K., Vilhjálmsson, B.J., Finucane, H.K., Salem, R.M., et al. (2015). Efficient Bayesian mixed-model analysis increases association power in large cohorts. *Nature Genetics* 47(3)**,** 284-290. doi: 10.1038/ng.3190.
22. Lu, Y.C., Day, F.R., Gustafsson, S., Buchkovich, M.L., Na, J.B., Bataille, V., et al. (2016). New loci for body fat percentage reveal link between adiposity and cardiometabolic disease risk. *Nature Communications* 7(1)**,** 10495. doi: 10.1038/ncomms10495.
23. Mariosa, D., Beard, J.D., Umbach, D.M., Bellocco, R., Keller, J., Peters, T.L., et al. (2017). Body Mass Index and Amyotrophic Lateral Sclerosis: A Study of US Military Veterans. *American Journal of Epidemiology* 185(5)**,** 362-371. doi: 10.1093/aje/kww140.
24. Nelson, L.M., Matkin, C., Longstreth, J.W.T., and McGuire, V. (2000a). Population-Based Case-Control Study of Amyotrophic Lateral Sclerosis in Western Washington State. II. Diet. *American Journal of Epidemiology* 151(2)**,** 164-173. doi: 10.1093/oxfordjournals.aje.a010184.
25. Nelson, L.M., McGuire, V., Longstreth, J.W.T., and Matkin, C. (2000b). Population-Based Case-Control Study of Amyotrophic Lateral Sclerosis in Western Washington State. I. Cigarette Smoking and Alcohol Consumption. *American Journal of Epidemiology* 151(2)**,** 156-163. doi: 10.1093/oxfordjournals.aje.a010183.
26. Nicolas, A., Kenna, K.P., Renton, A.E., Ticozzi, N., Faghri, F., Chia, R., et al. (2018). Genome-wide Analyses Identify KIF5A as a Novel ALS Gene. *Neuron* 97(6)**,** 1268-1283.e1266. doi: 10.1016/j.neuron.2018.02.027.
27. Nikpay, M., Goel, A., Won, H.-H., Hall, L.M., Willenborg, C., Kanoni, S., et al. (2015). A comprehensive 1000 Genomes-based genome-wide association meta-analysis of coronary artery disease. *Nature Genetics* 47(10)**,** 1121-1130. doi: 10.1038/ng.3396.
28. Noyce, A.J., Kia, D.A., Hemani, G., Nicolas, A., Price, T.R., De Pablo-Fernandez, E., et al. (2017). Estimating the causal influence of body mass index on risk of Parkinson disease: A Mendelian randomisation study. *PLoS Medicine* 14(6)**,** e1002314. doi: 10.1371/journal.pmed.1002314.
29. O'Reilly, É.J., Wang, H., Weisskopf, M.G., Fitzgerald, K.C., Falcone, G., McCullough, M.L., et al. (2013). Premorbid body mass index and risk of amyotrophic lateral sclerosis. *Amyotrophic Lateral Sclerosis and Frontotemporal Degeneration* 14(3)**,** 205-211. doi: 10.3109/21678421.2012.735240.
30. O'Reilly, E.J., Wang, M., Adami, H.O., Alonso, A., Bernstein, L., van den Brandt, P., et al. (2018). Prediagnostic body size and risk of amyotrophic lateral sclerosis death in 10 studies. *Amyotroph Lateral Scler Frontotemporal Degener* 19(5-6)**,** 396-406. doi: 10.1080/21678421.2018.1452944.
31. Okbay, A., Beauchamp, J.P., Fontana, M.A., Lee, J.J., Pers, T.H., Rietveld, C.A., et al. (2016). Genome-wide association study identifies 74 loci associated with educational attainment. *Nature* 533(7604)**,** 539-542. doi: 10.1038/nature17671.
32. Paternoster, L., Tilling, K., and Davey Smith, G. (2017). Genetic epidemiology and Mendelian randomization for informing disease therapeutics: Conceptual and methodological challenges. *PLoS Genetics* 13(10)**,** e1006944. doi: 10.1371/journal.pgen.1006944.
33. Peter, R.S., Rosenbohm, A., Dupuis, L., Brehme, T., Kassubek, J., Rothenbacher, D., et al. (2017). Life course body mass index and risk and prognosis of amyotrophic lateral sclerosis: results from the ALS registry Swabia. *European Journal of Epidemiology* 32(10)**,** 901-908. doi: 10.1007/s10654-017-0318-z.
34. Purcell, S., Neale, B., Todd-Brown, K., Thomas, L., Ferreira, M.A.R., Bender, D., et al. (2007). PLINK: A Tool Set for Whole-Genome Association and Population-Based Linkage Analyses. *American Journal of Human Genetics* 81(3)**,** 559-575. doi: 10.1086/519795.
35. Randall, J.C., Winkler, T.W., Kutalik, Z., Berndt, S.I., Jackson, A.U., Monda, K.L., et al. (2013). Sex-stratified Genome-wide Association Studies Including 270,000 Individuals Show Sexual Dimorphism in Genetic Loci for Anthropometric Traits. *PLoS Genetics* 9(6)**,** e1003500. doi: 10.1371/journal.pgen.1003500.
36. Rietveld, C.A., Medland, S.E., Derringer, J., Yang, J., Esko, T., Martin, N.W., et al. (2013). GWAS of 126,559 Individuals Identifies Genetic Variants Associated with Educational Attainment. *Science* 340(6139)**,** 1467.
37. Saxena, R., Hivert, M.-F., Langenberg, C., Tanaka, T., Pankow, J.S., Vollenweider, P., et al. (2010). Genetic variation in GIPR influences the glucose and insulin responses to an oral glucose challenge. *Nature Genetics* 42(2)**,** 142-148. doi: 10.1038/ng.521.
38. Scarmeas, N., Shih, T., Stern, Y., Ottman, R., and Rowland, L.P. (2002). Premorbid weight, body mass, and varsity athletics in ALS. *Neurology* 59**,** 773-775.
39. Scott, R.A., Scott, L.J., Mägi, R., Marullo, L., Gaulton, K.J., Kaakinen, M., et al. (2017). An Expanded Genome-Wide Association Study of Type 2 Diabetes in Europeans. *Diabetes* 66(11)**,** 2888-2902. doi: 10.2337/db16-1253.
40. Sheehan, N.A., Didelez, V., Burton, P.R., and Tobin, M.D. (2008). Mendelian randomisation and causal inference in observational epidemiology. *PLoS Medicine* 5(8)**,** e177. doi: 10.1371/journal.pmed.0050177.
41. Shungin, D., Winkler, T.W., Croteau-Chonka, D.C., Ferreira, T., Locke, A.E., Magi, R., et al. (2015). New genetic loci link adipose and insulin biology to body fat distribution. *Nature* 518(7538)**,** 187-196. doi: 10.1038/nature14132.
42. Sleiman, P.M., and Grant, S.F. (2010). Mendelian randomization in the era of genomewide association studies. *Clinical Chemistry* 56(5)**,** 723-728. doi: 10.1373/clinchem.2009.141564.
43. Splansky, G.L., Corey, D., Yang, Q., Atwood, L.D., Cupples, L.A., and Benjamin, E.J. (2007). The Third Generation Cohort of the National Heart, Lung, and Blood Institute’s Framingham Heart Study: design, recruitment, and initial examination. *American Journal of Epidemiology* 165(11)**,** 1328-1335. doi: 10.1093/aje/kwm021.
44. Sudlow, C., Gallacher, J., Allen, N., Beral, V., Burton, P., Danesh, J., et al. (2015). UK Biobank: An Open Access Resource for Identifying the Causes of a Wide Range of Complex Diseases of Middle and Old Age. *PLoS Medicine* 12(3)**,** e1001779. doi: 10.1371/journal.pmed.1001779.
45. Sutedja, N.A., van der Schouw, Y.T., Fischer, K., Sizoo, E.M., Huisman, M.H.B., Veldink, J.H., et al. (2011). Beneficial vascular risk profile is associated with amyotrophic lateral sclerosis. *Journal of Neurology, Neurosurgery and Psychiatry* 82(6)**,** 638-642. doi: 10.1136/jnnp.2010.236752.
46. The 1000 Genomes Project Consortium (2015). A global reference for human genetic variation. *Nature* 526(7571)**,** 68-74. doi: 10.1038/nature15393.
47. The International Consortium for Blood Pressure Genome-Wide Association Studies. (2011). Genetic variants in novel pathways influence blood pressure and cardiovascular disease risk. *Nature* 478(7367)**,** 103-109. doi: 10.1038/nature10405.
48. The Tobacco and Genetics Consortium (2010). Genome-wide meta-analyses identify multiple loci associated with smoking behavior. *Nature Genetics* 42(5)**,** 441-447. doi: 10.1038/ng.571.
49. Thomas, D.C., Lawlor, D.A., and Thompson, J.R. (2007). re: Estimation of bias in nongenetic observational studies using “Mendelian triangulation” by Bautista et al. *Annals of Epidemiology* 17(7)**,** 511-513. doi: 10.1016/j.annepidem.2006.12.005.
50. Veldink, J.H., Kalmijn, S., Groeneveld, G.J., Titulaer, M.J., Wokke, J.H., and van den Berg, L.H. (2005). Physical activity and the association with sporadic ALS. *Neurology* 64(2)**,** 241-245. doi: 10.1212/01.wnl.0000149513.82332.5c.
51. Visscher, P.M., Wray, N.R., Zhang, Q., Sklar, P., McCarthy, M.I., Brown, M.A., et al. (2017). 10 Years of GWAS Discovery: Biology, Function, and Translation. *American Journal of Human Genetics* 101(1)**,** 5-22. doi: 10.1016/j.ajhg.2017.06.005.
52. Walford, G.A., Gustafsson, S., Rybin, D., Stančáková, A., Chen, H., Liu, C.-T., et al. (2016). Genome-Wide Association Study of the Modified Stumvoll Insulin Sensitivity Index Identifies BCL2 and FAM19A2 as Novel Insulin Sensitivity Loci. *Diabetes* 65(10)**,** 3200-3211. doi: 10.2337/db16-0199.
53. Welter, D., MacArthur, J., Morales, J., Burdett, T., Hall, P., Junkins, H., et al. (2014). The NHGRI GWAS Catalog, a curated resource of SNP-trait associations. *Nucleic Acids Research* 42(D1)**,** D1001-D1006. doi: 10.1093/nar/gkt1229.
54. Wheeler, E., Leong, A., Liu, C.-T., Hivert, M.-F., Strawbridge, R.J., Podmore, C., et al. (2017). Impact of common genetic determinants of Hemoglobin A1c on type 2 diabetes risk and diagnosis in ancestrally diverse populations: A transethnic genome-wide meta-analysis. *PLoS Medicine* 14(9)**,** e1002383. doi: 10.1371/journal.pmed.1002383.
55. Willer, C.J., Schmidt, E.M., Sengupta, S., Peloso, G.M., Gustafsson, S., Kanoni, S., et al. (2013). Discovery and refinement of loci associated with lipid levels. *Nature Genetics* 45(11)**,** 1274–1283. doi: 10.1038/ng.2797.
56. Wood, A.R., Jonsson, A., Jackson, A.U., Wang, N., van Leewen, N., Palmer, N.D., et al. (2017). A genome-wide association study of IVGTT-based measures of first phase insulin secretion refines the underlying physiology of type 2 diabetes variants. *Diabetes* 66(8)**,** 2296-2309.
57. Wu, Y., Zheng, Z., Visscher, P.M., and Yang, J. (2017). Quantifying the mapping precision of genome-wide association studies using whole-genome sequencing data. *Genome Biology* 18(1)**,** 86. doi: 10.1186/s13059-017-1216-0.
58. Yang, J., Ferreira, T., Morris, A.P., Medland, S.E., Genetic Investigation of, A.T.C., Replication, D.I.G., et al. (2012). Conditional and joint multiple-SNP analysis of GWAS summary statistics identifies additional variants influencing complex traits. *Nature Genetics* 44**,** 369-375. doi: 10.1038/ng.2213.
59. Yengo, L., Sidorenko, J., Kemper, K.E., Zheng, Z., Wood, A.R., Weedon, M.N., et al. (2018). Meta-analysis of genome-wide association studies for height and body mass index in ~700000 individuals of European ancestry. *Human Molecular Genetics***,** 10.1093/hmg/ddy1271. doi: 10.1093/hmg/ddy271.
